# TIM4+macrophages suppress the proinflammatory response to maintain the chronic alveolar echinococcosis infection

**DOI:** 10.3389/fcimb.2025.1600686

**Published:** 2025-06-18

**Authors:** Liang Wang, Yumei Liu, Yuyu Ma, Xuan Zhou, Maidinaimu Aibibula, Xue Zhang, Hui Zhao, Jinping Zhou, Fengming Tian, Xiumin Ma

**Affiliations:** ^1^ Institute of Medical Sciences of Xinjiang Medical University, Department of Medical Laboratory Center, Tumor Hospital Affiliated to Xinjiang Medical University, Urumqi, China; ^2^ Clinical Laboratory Center, Fifth Affiliated Hospital of Xinjiang Medical University, Urumqi, China; ^3^ Clinical Laboratory Center, Hospital of Traditional Chinese Medicine affiliated to Xinjiang Medical University, Urumqi, China; ^4^ State Key Laboratory of Pathogenesis, Prevention and Treatment of High Incidence Diseases in Central Asia, First Affiliated Hospital of Xinjiang Medical University, Urumqi, China; ^5^ Postdoctoral Research Workstation of Tumor Hospital Affiliated to Xinjiang Medical University, Urumqi, China

**Keywords:** *Echinococcus multilocularis*, Tim-4, parasite infection, immunity, macrophage, liver fibrosis

## Abstract

**Background:**

Alveolar echinococcosis (AE), a severe zoonotic disease predominantly endemic to pastoral regions, is characterized by hepatic parasitic lesions caused by *Echinococcus multilocularis*.

**Methods:**

This study investigated the role of T-cell immunoglobulin and mucin domain-4 (TIMD4/Tim-4) in patients with hepatic AE. In total, 129 patients were enrolled from the First Affiliated Hospital of Xinjiang Medical University between 1 March 2018 and 1 March 2021. Histological, genetic, and serological tests were employed to evaluate Tim-4 and inflammatory cytokine expression. The liver immune microenvironment at the middle and late stages of mice infected with *E. multilocularis* was established *in vitro* to assess cytokine dynamics and liver fibrosis biomarkers.

**Results:**

Clinical analysis revealed the upregulation of Tim-4 within the hepatic lesions of patients with AE, with its expression spatially localized to macrophage-enriched regions and functionally linked to extracellular inflammatory modulation. Meanwhile, the liver tissues of the patients had characteristic pathological changes in the vesicles and progressive fibrotic remodeling, concurrent with a significant suppression of proinflammatory cytokine activity. Tim-4+ macrophages inhibited the release of proinflammatory cytokines at the middle and late stages of *E. multilocularis* infection to maintain immune tolerance, and inhibition of Tim-4 expression may even reverse the level of liver fibrosis *in vitro*.

**Conclusions:**

Tim-4 attenuated the predominant proinflammatory response, thereby facilitating immune evasion by *E. multilocularis*. Notably, inhibition of Tim-4 in macrophages not only restored the inflammatory balance but also significantly reversed hepatic fibrotic progression.

## Highlights

- Tim-4 emerged as a potential target associated with *E. multilocularis* infection in patients with hepatic AE.
- Tim-4 attenuated the predominant proinflammatory response, thereby facilitating immune evasion by *E. multilocularis*. Notably, inhibition of Tim-4 in macrophages not only restored inflammatory balance but also significantly reversed hepatic fibrotic progression.

## Introduction

Cystic echinococcosis (CE) and alveolar echinococcosis (AE), representing the two predominant clinical manifestations of human echinococcosis worldwide, are caused by the tapeworms *Echinococcus granulosus* and *Echinococcus multilocularis*, respectively. Humans usually get infected with both diseases accidentally as intermediate hosts when they ingest eggs of *Echinococcus* spp. transmitted from infected definitive hosts or come into contact with a contaminated environmental source. Hepatic involvement predominates in the clinical manifestations of both diseases, with the liver serving as the primary target organ for larval cyst development. Present evidence indicates that the Chinese mainland experiences the highest prevalence of echinococcosis (Wang et al., 2021). AE is a lethal disease with a 10-year fatality rate of 94% for untreated or inadequately managed patients (Ma et al., 2023). Echinococcosis has been listed as one of the 20 neglected tropical diseases recognized by the World Health Organization (WHO) and is prioritized for control efforts (Casulli, 2020).

In experimental intra-hepatic *E. multilocularis* infection, depletion of macrophages during the first 6 weeks facilitates parasite establishment and perhaps also parasite development after laminated layer deployment (Wang et al., 2020). Macrophage accumulation at infection sites can be brought about by the recruitment of their monocyte precursors. Macrophages proliferate at the site and this process is marked in type 2-like contexts such as helminth infections (Jenkins and Allen, 2021). The contributions of macrophage recruitment and proliferation to macrophage accumulation in chronic *Echinococcus* infections should attract attention. As mentioned, evidence is growing for strong regulatory responses in echinococcosis (Gottstein et al., 2017; Pang et al., 2014; Fratini et al., 2020). Since attaining an overall lack of pro-inflammatory signals often requires active downregulation, *Echinococcus* may have developed the capacity to deliver anti-inflammatory signals to host immune cells, and the host responds through parasite-derived immune regulators (Díaz et al., 2023). Our previous study also showed that inflammatory cells were recruited to the liver during the initial parasite infection, which released cytokines mainly to fight against the parasitic infection, and later a large number of anti-inflammatory cytokines were released to promote liver repair (Tian et al., 2021). The pathophysiological progression of *E. multilocularis* infection involves dynamic immunological adaptation characterized by the establishment of immune tolerance, concomitant with progressive hepatic fibrogenesis. This process is mechanistically linked to programmed hepatocyte death (encompassing both apoptosis and necroptosis), ultimately resulting in the disruption of hepatic immune homeostasis and compromised regenerative capacity of the liver parenchyma.

T-cell immunoglobulin and mucin domain-4 (TIMD4/Tim-4) are characterized as phosphatidylserine (PtdSer) receptors expressed on mouse antigen-presenting cells. Its critical role in the engulfment of PtdSer-expressing apoptotic bodies by macrophages and dendritic cells has also been confirmed in humans (Miyanishi et al., 2007). In human cells, Tim-4 is restricted to liver Kupffer cells, tingible-body macrophages, and splenic white pulp macrophages (Dorfman et al., 2010), but data on its expression in human immune cells remains very scant. Chow et al. found Tim-4 expression on liver Kupffer cells, but no evidence showed Tim-4 expression on steady-state murine or human circulating monocytes (Chow et al., 2021). This study focused on the immune role of Tim-4+ macrophages, especially their immunogenic role during the chronic progression of hepatic AE.

## Materials and methods

### Biological function of TIMD4 in patients with AE

Long non-coding RNA (lncRNA) and mRNA expression profiles comprising paired hepatic tissue samples (n=12, perilesional vs. distal hepatic tissues) in patients with hepatic alveolar echinococcosis (HAE) were obtained from the GEO database (https://www.ncbi.nlm.nih.gov/geo/, dataset GSE124362). Agilent Feature Extraction software (version 11.0.1.1) was used to process raw array images. The Gene Spring GX v11.5.1 package was used for quantile normalization and data processing. After quantile normalization of the original data, qualified lncRNA and mRNA profiles were selected for subsequent analysis. The clean datasets from the above database were exported via R Studio (version 3.6.3) for differential expression analysis. The threshold of the volcano map was |logFC|> 1 and P <0.05. Upregulated genes specifically associated with alveolar echinococcosis pathogenesis were filtered under stringent thresholds (logFC>1, P<0.05).

Cytoscape software was used to cluster differentially expressed genes. The key gene Tim-4 was selected according to the expression of differentially upregulated genes and related literature. Gene Ontology (GO) was performed based on the *Tim-4* expression-relevant genes. Specifically, the P-value of the enrichment result is represented by color, and the gene number is represented by the bubble size. The cluster Profiler toolkit was used to obtain the correlation of target genes and to visualize the related graphs.

To investigate the abundance of infiltration of immune cells and the correlation between the *Tim-4* gene and the abundance of infiltration of immune cells in the livers of patients with AE, the Wilcoxon rank sum and Spearman rank correlation tests were used. Statistical analysis and visualization were performed in R studio (version 3.6.3), involving the R package GSVA (version 1.34.0).

### Patients

A total of 129 patients were enrolled from the First Affiliated Hospital of Xinjiang Medical University (1 March 2018 – 1 March 2021), comprising patients with HAE (n=33) and healthy controls (n=96). The diagnosis of patients with hepatic AE (patients were confirmed by B-mode ultrasonography, then underwent liver cystic hydatidosis partial hepatectomy, and their biopsy specimens were collected) was made using the classification diagnostic criteria formulated by the WHO’s unofficial echinococcosis working group (Tian et al., 2020). Paired liver lesion tissue and normal tissue were obtained from each patient with HAE who underwent a biopsy of normal hepatic tissue (the normal hepatic tissue 2 cm away from the lesion) was used as the control group and hepatic tissue adjacent to the lesion, which was not directly part of the lesion, as the case group (within 2 cm of the lesion) (Zhang et al., 2016). Furthermore, 3 ml of peripheral blood was collected from patients and healthy controls. The peripheral blood was centrifuged at 3,000 rpm for 10 min at RT; the hemocytes were prepared for peripheral blood mononuclear cell (PBMC) extraction. The serum was used for Enzyme linked immunosorbent assay (ELISA). The study protocols were approved by the ethics committee of the First Affiliated Hospital of Xinjiang Medical University (No. 20170214-106) and informed consent was received from all patients.

### Cells

The mouse hepatic stellate cell line (mHSC) and mouse macrophage cell line (RAW264.7) were purchased from the Bena Culture Collection (ATCC, VA, USA). The mHSC was cultured in DMEM supplemented with 10% FBS and 1% penicillin-streptomycin solution. RAW264.7 was cultured with RAW 264.7 cell-specific medium (Procell, Wuhan, China) at 37°C in a 5% CO_2_ humidified atmosphere. The mHSC and RAW264.7 cell lines were indirectly cocultured by a cell culture chamber (LABSELECT, Anhui, China). To establish stable cell lines, lentiviral vectors to increase or decrease Tim-4 expression levels and a vector-only control were purchased from Hanbio (Shanghai, China). Lentiviral particles were transduced into the RAW264.7 cell line, followed by puromycin screening for 2 weeks. Expression of TRIM36 was identified by qRT-PCR.

### Animals

Eight-week-old female Balb/c mice (n=10) were purchased from the Animal Laboratory Center at Xinjiang Medical University (Urumqi, China) and infected with *E. multilocularis*. The method of the infection model was described in our previous study (Tian et al., 2021) and five mice were used as the control. The mice were raised in an air-conditioned room with a 12-hour light/dark cycle and provided with food and water. The mice were euthanized using cervical dislocation at 180 days after infection. The infected liver tissue was thoroughly crushed with PBS to form a homogenate (volume ratio of tissue and PBS=1:9), which was repeatedly freeze-thawed in -80°C and filtered using 0.45 μm and 0.33 μm filters as a stimulant to intervene cell lines. The mice were treated according to the guidelines of the Institutional Animal Care, with protocols approved by the Institutional Animal Use and Care Committee of Xinjiang Medical University (No. 20170214-106).

### Liver histopathological observation

Hepatic specimens were fixed in 4% formalin and embedded in paraffin. Non-consecutive 3 µm sections were stained with hematoxylin-eosin (HE) and Mason’s Trichrome (according to the manufacturer’s protocol), and the pathological change and fibrosis were observed under a microscope.

For HE staining, the sections were dehydrated, stained with hematoxylin (Baoman Biotechnology, Shanghai, China) for 3 min, and washed with water for 30 s. Then, the sections were differentiated by 1% hydrochloric acid and alcohol, washed with water for 30 s, stained with eosin at room temperature (RT) for approximately 3 min, dehydrated by graded ethanol, and cleared by xylene once for approximately 1 min.

For Masson staining, sections were dewaxed in water, stained with hematoxylin for 3 minutes, rinsed with water, followed by hydrochloric acid for 5 s, rinsed with water again, and stained with ammonia for several minutes. Then, garnet magenta (10 s), 12-molybdenum-phosphate solution (3 min), and green staining solution (4 min) were successively dyed. The slices were washed with water and dried, sealed with neutral adhesive, and observed under a microscope. Image J was used to analyze 5 discontinuous random organization fields.

### IHC and IF

The tissue sections were dehydrated, processed for heat-mediated antigen-retrieval using Tris-EDTA buffer for 15 minutes (ZSGB-BIO, Beijing), and then cooled at room temperature (RT). Sections were blocked with 10% goat serum for 1 h and then incubated with primary antibodies at 4°C overnight (rabbit anti-human Tim-4, 1:500, Abcam, Cambridge, UK; rabbit anti-human CK-18, Proteintech, Wuhan, China). Sections were washed with PBS and incubated for 2 h with a secondary antibody [goat anti-rabbit F(ab’)2-HRP]. Staining was developed using a 3, 3’ Diaminobenzidine (DAB) substrate kit (Abcam) according to the manufacturer’s instructions. Staining was then assessed at 200× or 400× magnification in a total of 3–5 fields/section/sample using cellSens Dimension software (Olympus) for computerized quantification, and the results were expressed as the intensity of positive staining per field.

For immunofluorescence, the procedure was similar to IHC. The sections were incubated with primary antibodies at 4°C overnight (rabbit anti-human Tim-4, 1:250, mouse anti-human CD68, 1:1000, Abcam, Cambridge; rabbit anti-human CK-18, Proteintech, Wuhan, China). The next day, the sections were washed with PBS and incubated for 2 h with secondary antibody (goat anti-mouse IgG H&L Alexa Fluor^®^488, 1:200; goat anti-rabbit IgG H&L Alexa Fluor^®^647, 1:200). DAPI was then added to the sections in the dark. Finally, we observed the sections under a confocal microscope.

### PBMC extraction

Five ml of TBD lymphocyte isolation solution (Haoyang, Tianjin, China) was used to hold the diluted blood (fresh human peripheral blood was mixed with the same volume of PBS), and then centrifuged at 2,000 rpm with controlled acceleration/deceleration rates for 30 min at 4°C. The buffy coat interface was aspirated using angled pipettes. Cells were washed with PBS twice and prepared for RNA extraction.

### qRT-PCR and ELISA

Total RNA was extracted from PBMCs and cell lines using a Trizol™ isolation kit (Takara Bio, Dalian, China), subsequently converted to cDNA using a PrimeScript reagent kit with gDNA Eraser (Takara Bio, Dalian, China), and subjected to real-time PCR using SYBR Premix Ex Taq II (Takara Bio). The primers (**Table 1**) were synthesized by Sangon Biotech (Shanghai, China). The details are shown in **Table 1**. Real-time PCR was conducted on the ABI Prism 7500 Sequence Detection System (BioRad, Life Science Research, Hercules, CA, USA). The PCR conditions were as follows: one cycle at 95°C for 30 s, 40 cycles at 95°C for 5 s, and at 61°C for 30 min. All samples were run in triplicate. Relative mRNA abundances were determined using the 2^−ΔΔCt^ method using the GAPDH gene to normalize. Serum concentrations of Tim-4 were determined using an ELISA kit (Jianglai Biotech, Shanghai, China) following the manufacturer’s protocol.

**Table 1:** Primer sequence.

Gene	Primers(5’→3’)
hTim-4	Forward:GTACTGCTGCCGCATAGAAGT
	Reverse:TTGTTGTCATTTGTCGGGTGG
hIL-6	Forward:ACTCACCTCTTCAGAACGAATTG
	Reverse:CCATCTTTGGAAGGTTCAGGTTG
hTNF-α	Forward:GAGGCCAAGCCCTGGTATG
	Reverse:CGGGCCGATTGATCTCAGC
hIL-1β	Forward:TTCGACACATGGGATAACGAGG
	Reverse:TTTTTGCTGTGAGTCCCGGAG
hIL-18	Forward:TCTTCATTGACCAAGGAAATCGG
	Reverse:TCCGGGGTGCATTATCTCTAC
hGAPDH	Forward:CATCCACTGGTGCTGCCAAGGCTGT
	Reverse:ACA ACCTGGTCCTCAGTGTAGCCCA
mTim-4	Forward:CCGGTGACTTTGCCTTGTCAT
	Reverse:CTCTGCATTGCACTTGGAATTG
mIL-6	Forward:CTGCAAGAGACTTCCATCCAG
	Reverse:AGTGGTATAGACAGGTCTGTTGG
mIL-18	Forward:GTGAACCCCAGACCAGACTG
	Reverse:CCTGGAACACGTTTCTGAAAGA
mIL-1β	Forward:GAAATGCCACCTTTTGACAGTG
	Reverse:TGGATGCTCTCATCAGGACAG
mTNF-α	Forward:CAGGCGGTGCCTATGTCTC
	Reverse:CGATCACCCCGAAGTTCAGTAG
mIL-10	Forward:ATAAGAGCAAGGCAGTGGAGC
	Reverse:GGCCTTGTAGACACCTTGGTC
mMMP9	Forward:GGACCCGAAGCGGACATTG
	Reverse:CGTCGTCGAAATGGGCATCT
mCaspase3	Forward:CTCGCTCTGGTACGGATGTG
	Reverse:TCCCATAAATGACCCCTTCATCA
mα-SMA	Forward:GTCCCAGACATCAGGGAGTAA
	Reverse:TCGGATACTTCAGCGTCAGGA
mTGF-β	Forward:AGCTGCGCTTGCAGAGATTA
	Reverse:GACAGCCACTCAGGCGTATC
mCol1α1	Forward:TAAGGGTCCCCAATGGTGAGA
	Reverse:GGGTCCCTCGACTCCTACAT
mGAPDH	Forward:TGGTGAAGCAGGCATCTGAG
	Reverse:TGAAGTCGCAGGAGACAACC

### Statistical analysis

The quantitative analysis of the morphology results was carried out using Image J. Data were analyzed by SPSS 21.0 (IBM, Chicago, IL, USA) or GraphPad Prism 8.0 software (GraphPad Software, San Diego, CA, USA). Results were expressed as means ± standard error of the mean (SEM). Differences between groups were analyzed according to variable distribution using *t*-test/Mann-Whitney for two groups or ANOVA/Kruskal–Wallis (with Bonferroni or Dunn posttests, respectively) for the comparison between multiple groups. A *P*-value <0.05 was considered statistically significant.

## Results

### Tim-4 was mainly expressed in M2-like immunosuppressive macrophages in patients with hepatic AE

**Figure 1:**
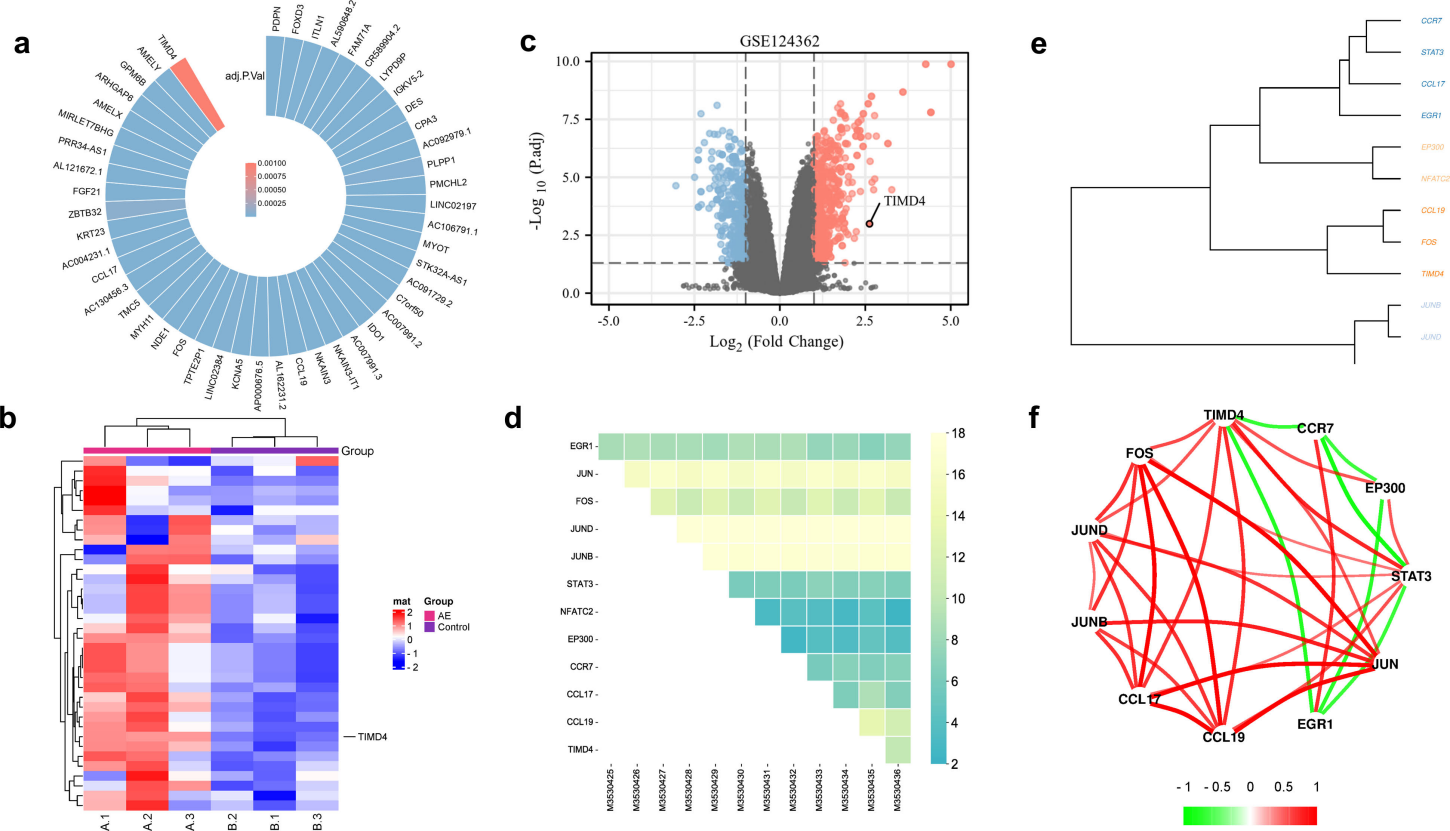
*Tim-4* was highly expressed in liver lesions in patients with hepatic AE. **(a)**. Upregulated genes in patients with hepatic AE; **(b, c)**. TIMD4 was highly expressed in patients with hepatic AE; **(d)** Top 12 upregulated genes in patients with hepatic AE; **(e, f)**. Correlation analysis between upregulated genes in patients with hepatic AE.

Analysis of the GSE124362 dataset (n=12, comprising paired perilesional and distal hepatic tissues from six patients with hepatic AE) revealed a stable upregulation of TIMD4 in hepatic lesions (**Figures 1a–c**) and identified the top 12 significantly upregulated genes (**Figures 1d–f**). Among them was EGR1, a key regulator of fibrotic scar formation (Wang et al., 2023). The main members of the activator protein-1 (AP-1) family include JUN, JUND, JUNB, and FOS, which orchestrate inflammatory factor-mediated vascular remodeling and cellular proliferation (Gao et al., 2021), and immunomodulatory markers CCL17/TIMD4, associated with M2-like macrophage immunosuppression (Kuninaka et al., 2022). CCL19 facilitates the migration of immune cells (Takagi-Kimura et al., 2022). Tim-4 has been positively correlated with M2-like macrophage-associated inflammatory factors and negatively correlated with fibrosis genes (**Figure 1f**).

**Figure 2:**
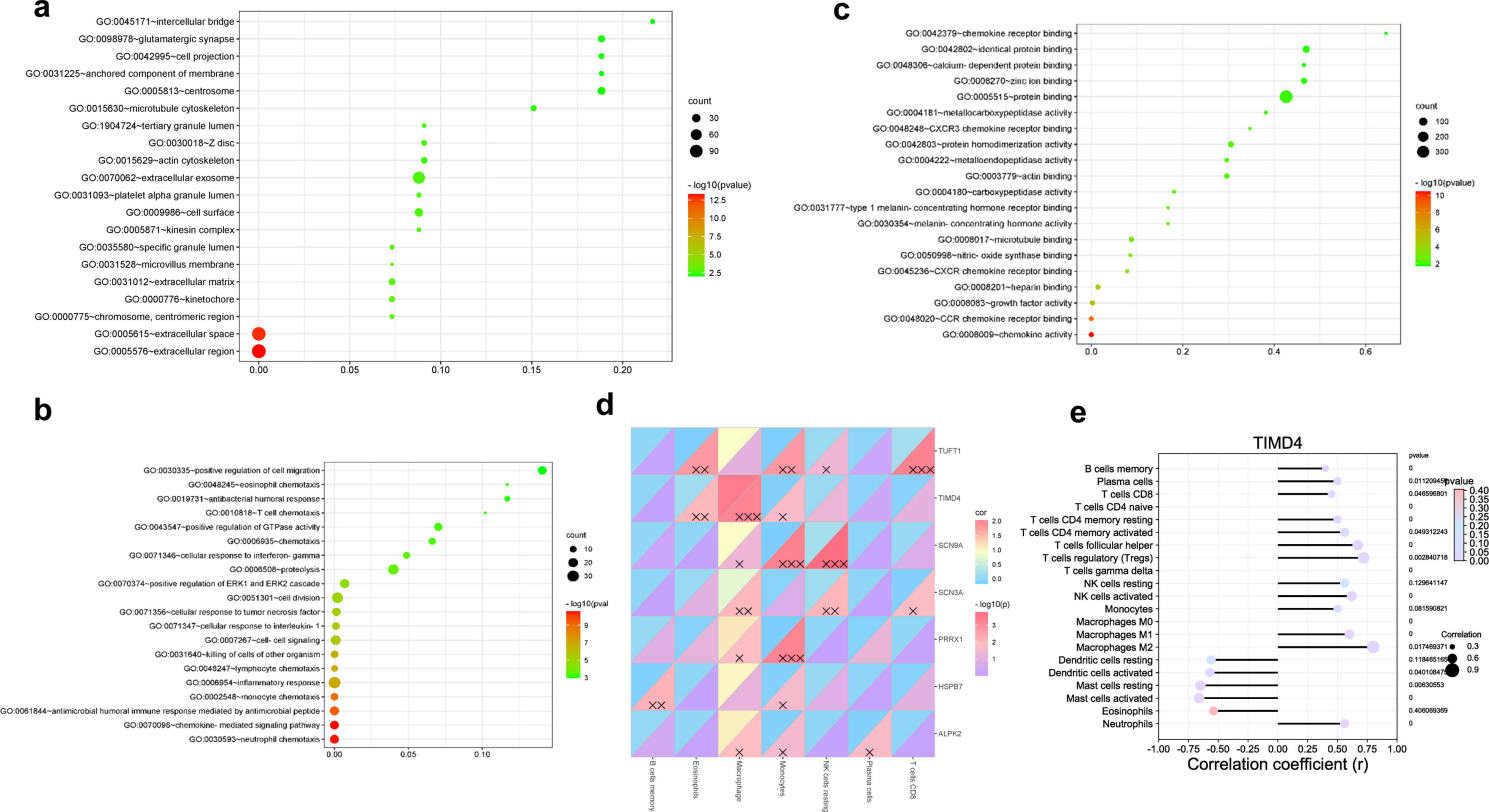
Tim-4 was mainly expressed in M2-like immunosuppressive macrophages in patients with hepatic AE. **(a–c)**. TIMD4 molecular function enrichment in patients with hepatic AE; **(d, e)**. Correlation analysis between TIMD4 and immune infiltration in patients with hepatic AE; X, *P*<0.05; XX, *P*<0.01; XXX, *P*<0.001.

Functional enrichment analysis of differentially upregulated genes via DAVID revealed that Tim-4 was mainly involved in the chemotaxis of extracellular inflammation (**Figures 2a–c**). Tim-4 was mainly expressed in M2-like immunosuppressive macrophages in patients with hepatic AE. The immuno-infiltration analysis showed that activated mast cells, γδT cells, M2-like macrophages, and activated NK cells were mainly positively correlated with hepatic AE (**Figures 2d, e**). These findings suggested that during *E. multilocularis* infection, a complex interplay occurs among multiple immune cell types within the patient’s hepatic tissue, presenting a complex immune microenvironment. Various immune cells collaborate to exert a synergistic immunosuppressive effect and jointly maintain liver immune tolerance, especially in the middle and late stages of *E. multilocularis* infection.

### Severe inflammatory change and advanced fibrosis of liver tissue in patients with hepatic AE

**Figure 3:**
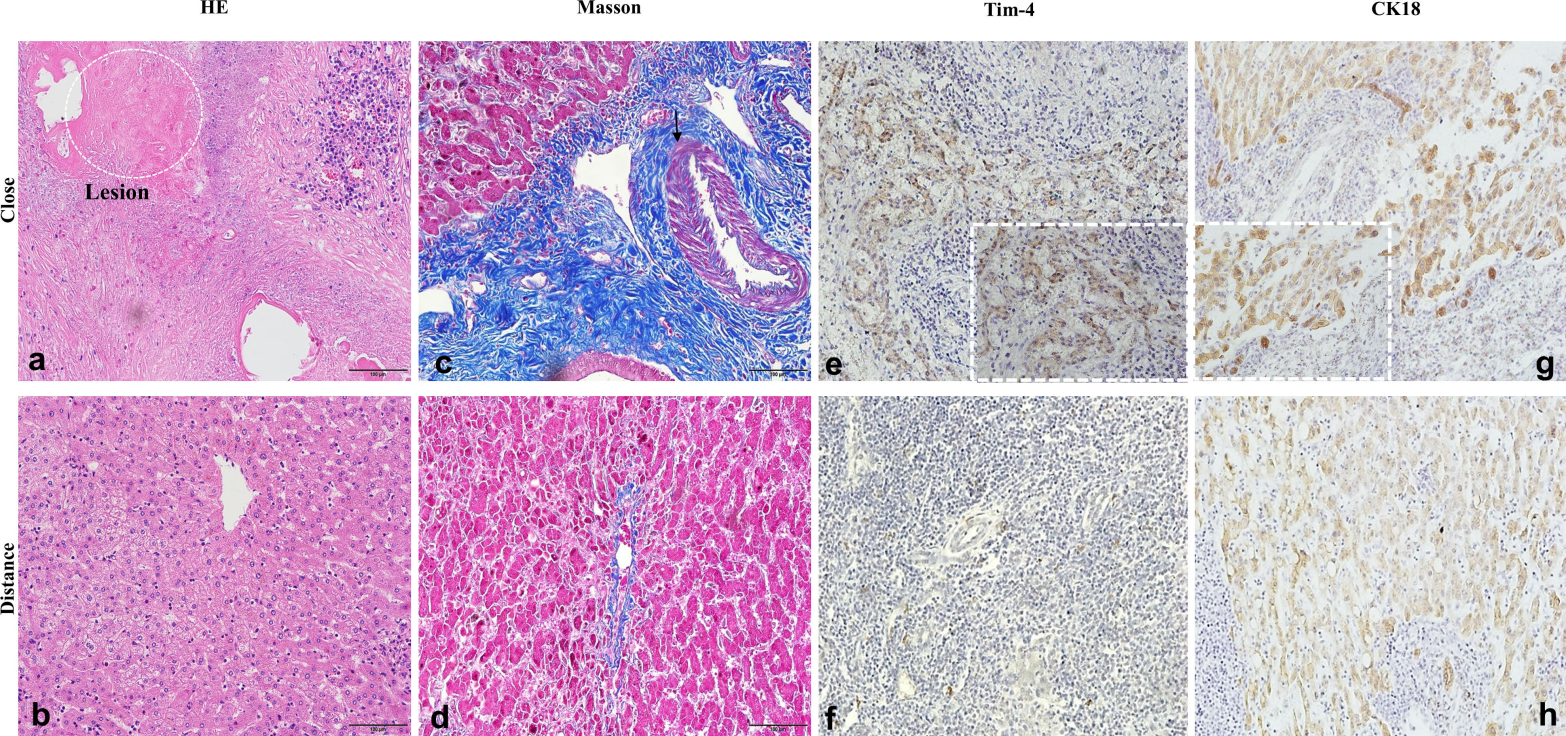
Severe inflammatory change and advanced fibrosis of liver tissue in patients with hepatic AE. **(a–d)** Inflammation and fibrosis of liver tissue in patients with AE (×20); **(e–h)** High expression of Tim-4 and CK18 in patients with hepatic AE (×20, ×40).

Histopathological analysis by HE staining revealed characteristic hepatic architectural alterations in the proximal liver tissues of patients with AE. The hepatic architecture was markedly disrupted with structural disorganization, accompanied by extensive immune cell infiltration surrounding the calcified liver tissue. There were vesicles of *E. multilocularis* similar to the mesenchyme, which was a typical pathological change due to *E. multilocularis* infection (**Figures 3a, b**). These calcification-associated parasitic vesicles demonstrated spatial colocalization with sustained immune cell accumulation, reflecting the chronic progression of the parasitic lesion. Masson staining demonstrated marked contrast in collagen deposition patterns between the hepatic zones in patients with hepatic AE. The distal parenchyma exhibited minimal perivascular collagen deposition with preserved lobular architecture, whereas proximal regions displayed extensive collagenous matrix deposition forming characteristic perilesional fibrotic capsules (**Figures 3c, d**). Tim-4 protein was highly expressed in the proximal tissue of liver lesions in patients with hepatic AE (**Figures 3e, f**). The positive expression of CK18 also suggested that the liver parenchymal cells were seriously damaged (**Figures 3g, h**).

### Tim-4 was highly expressed in infiltrating macrophages in the proximal liver tissue in patients with hepatic AE

**Figure 4:**
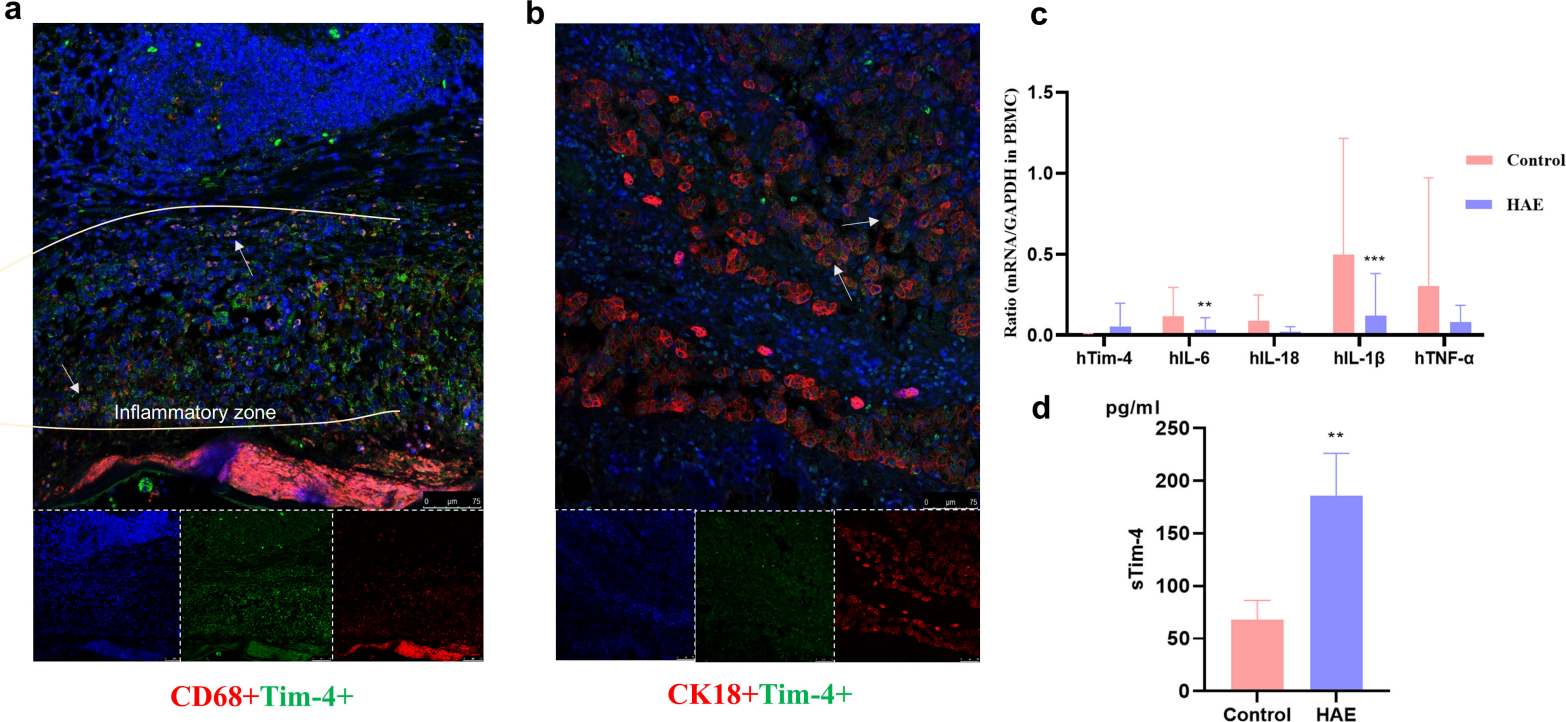
Tim-4 was highly expressed in infiltrating macrophages in the proximal liver tissue in patients with hepatic AE. **(a)** Tim-4 is highly expressed in infiltrating macrophages in the liver tissue of patients with AE in the Close group (×20). The white arrows indicate CD68+Tim-4+macrophages; **(b)** Tim-4 is highly expressed in the hepatocytes in the liver tissue of patients with AE in the Close group (×20). The white arrows indicate CK18+Tim-4+macrophages; **(c)** The mRNA expression of related inflammatory factors in the patients’ PBMCs; **(d)** Detection of sTim-4 expression in peripheral plasma by ELISA. **, P<0.01; ***, P<0.001.

Compared with the distal liver tissue of patients with hepatic AE, a large number of inflammatory cells infiltrated around the lesion to form a vague inflammatory cell zone (**Figure 4a**). CD68, a specific marker of human macrophages; CD68; and Tim-4 protein were co-expressed in the proximal lesion tissue. Tim-4 was highly expressed in hepatocytes in liver tissue of patients with AE in the lesion tissue (**Figure 4b**). Tim-4, as a secreted protein, mainly played an extracellular inflammatory inhibitory role and was expressed in liver cells, suggesting that Tim-4 was abnormally highly expressed in antigen-presenting (APC) cells in AE, which also further expressed on hepatocytes to further induce liver tissue and function disorders.

We analyzed the serum release of Tim-4 and the expression of inflammatory cytokines in the PBMCs of patients with hepatic AE and found that the proinflammatory cytokine expression of IL-1β, IL-18, IL-6, and TNF-α were lower than in healthy patients (*P*<0.05) (**Figure 4c**), while Tim-4 showed a high serum level (**Figure 4d**), which also suggested that Tim-4 mainly played an extracellular anti-inflammatory role.

### Inhibition of Tim-4 expression on macrophages even reversed the level of liver fibrosis *in vitro*

**Figure 5:**
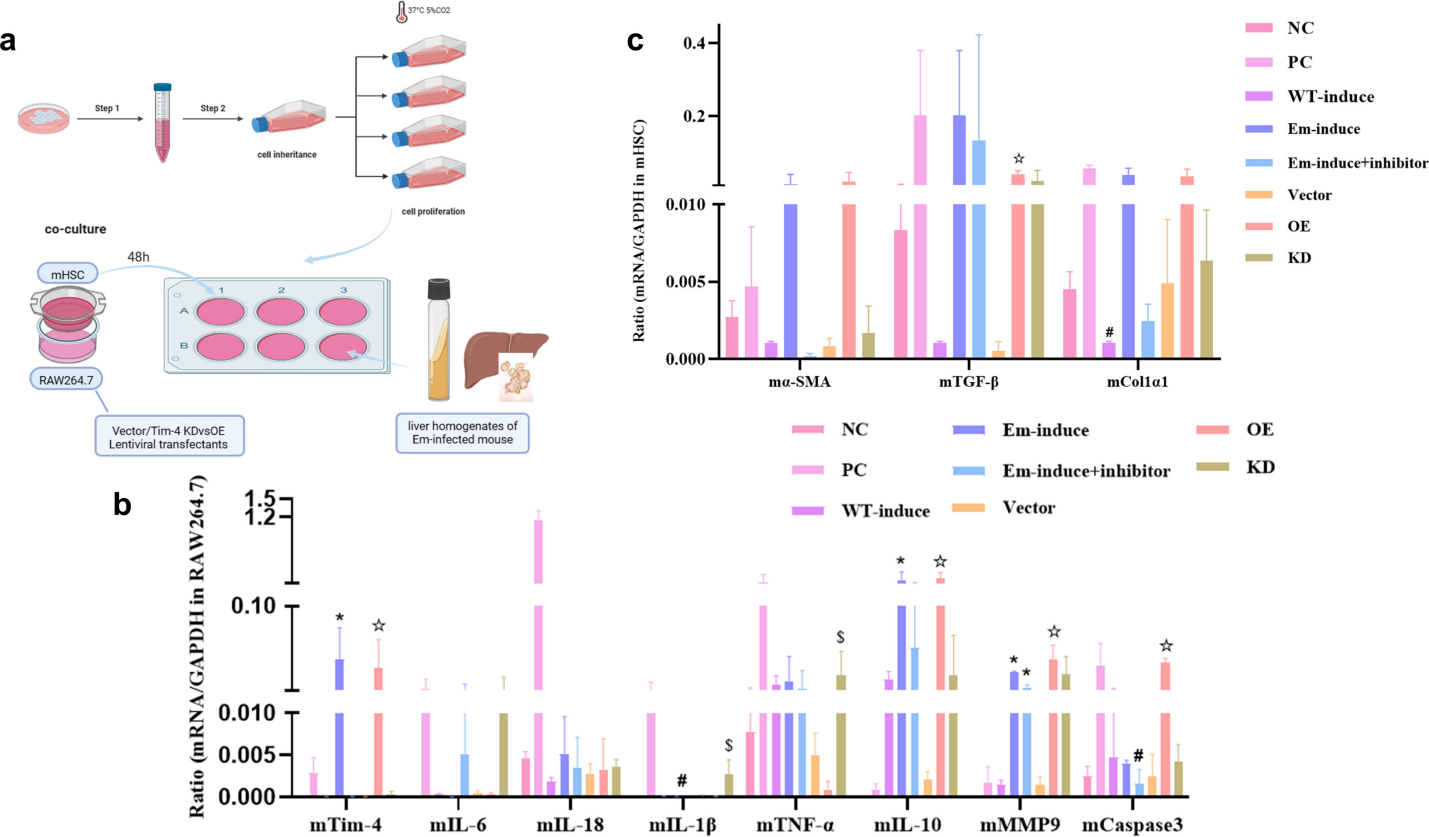
Inhibition of Tim-4 expression on macrophages even reversed the level of liver fibrosis *in vitro.* **(a)** Indirect co-culture models of the liver microenvironment in the middle and late stages of *E*. *multilocularis* infection; **(b)** mRNA expression of different cytokines; *, compared with the NC group, *P*<0.05; #, compared with the Tim-4 mAb group, *P*<0.05; ☆, compared with the Vector group, *P*<0.05; $, compared with the Tim-4 OE group, *P*<0.05; **(c)** mRNA expression of fibric cytokines; #, compared with the WT group, *P*<0.05; ☆, compared with the Vector group, *P*<0.05.

We established an indirect transwell co-culture model combining murine macrophage cell line (RAW264.7) and murine hepatic stellate cell line (mHSC) in a complete medium. The co-cultures were exposed to sterile-filtered liver homogenates derived from either wild-type (WT) controls or 90-day post-infection *E. multilocularis*-infected mice, and we then intervened with knockdown (KD) or overexpression (OE) of *Tim-4* siRNA constructs, respectively (**Figure 5a**). The results showed that the expression levels of proinflammatory factors such as IL-18, IL-1β, IL-6, and TNF-α were significantly increased in the PC group, among which IL-1β was significantly increased compared with the NC group (*P* < 0.05) (**Figure 5b**). The expression level of proinflammatory cytokines was significantly lower, while the expression level of IL-10, a classic inhibitory inflammatory cytokine, was significantly higher than that of the NC group (*P* < 0.05) (**Figure 5b**), suggesting that the inhibitory inflammatory cytokines were in a dominant expression state, and the overall cell environment was in a state of immunosuppression in the liver microenvironment of mice infected with *E. multilocularis*.

MMP9 is mainly involved in inflammation, tissue destruction, fibrosis, and other pathological changes, and the expression of MMP9 in the liver microenvironment of multicompartment *Echinococcus*-infected mice was significantly increased, with a statistical difference compared with the NC group (*P* < 0.05). In the state of inflammatory activation, the role of caspase3 as an apoptosis inducer was significantly amplified, and its activity level was notably higher than that observed in the Tim-4 inhibition group (*P* < 0.05) (**Figure 5b**). In our study, fibrosis-related cytokines produced by mHSCs also exhibited remarkable alterations in various cell model groups (**Figure 5c**). α-SMA, as a marker of liver fibrosis, is a classical marker of mHSC activation. Notably, within the liver microenvironment of mice infected with *E. multilocularis*, the activation status of mHSCs underwent a decline following the interference from Tim-4 mAb, and the expression level of Col1α1 also showed a similar expression trend to α-SMA. Compared with the NC group and the WT-induce group, the expression of TGF-β exhibited distinct patterns: it was notably upregulated in the PC and the Em-induce groups. Conversely, upon inhibition of *Tim-4*, the expression of TGF-β in macrophages was significantly downregulated. Though we constructed *Tim-4* overexpression and *Tim-4* downexpression groups, the expression patterns of fibrosis-related genes was basically consistent with those in the WT-induce, Em-induce, and Tim-4 mAb groups, and the TGF-β gene was significantly upregulated in the *Tim-4* overexpression group (compared with the empty vector group, the TGF-β gene was significantly upregulated) (*P* < 0.05). Therefore, we suggest that reduced Tim-4 expression on macrophages in the middle and late stages of *E. multilocularis* infection can alleviate or even reverse fibrosis levels.

## Discussion

A typical feature of AE is a tumor-like aggressive proliferation of the metacestodes. Hepatic involvement is dominant in AE, with approximately 90% of the alveolar cysts found in the liver (Ghasemirad et al., 2022). This metacestode proliferation not only infiltrates the adjacent host tissues to form pseudocystic lesions but also forms metastases in distant organs, with massive periparasitic infiltration of the host’s cells and fibrosis (Li et al., 2023). The therapeutic arsenal for AE is constrained. Partial hepatectomy remains the main curative approach but is only indicated when complete parasitic lesion removal is achievable (Akbulut et al., 2018; Joliat et al., 2023). The majority of the patients with AE (64.3%) received ABZ therapy, while only 32.1% underwent surgical resection combined with ABZ treatment, indicating that complete lesion resection was unachievable in most cases, particularly among immunocompromised patients with AE. It has been estimated that only one-third of patients with AE are eligible for potentially curative hepatic surgery due to the significant morbidity and mortality resulting from complications (Wen et al., 2019). Immune tolerance is one of the major hallmarks of *E. multilocularis* infection. During infection, multiple host immune cells interact with the parasites within hepatic tissues and form a granulomatous inflammatory microenvironment (Meng et al., 2022).

As previously reported (Tian et al., 2021), in the murine model of *E. multilocularis* infection, macrophage-mediated immune tolerance was established during the early infection phase. This likely contributes to the development of an immunosuppressive hepatic microenvironment, facilitating immune evasion by the parasite. In a cross-sectional study, it was reported (Lachenmayer et al., 2019) that of 131 patients with AE, 41 (31%) were immunosuppressed. Immune tolerance represents a defining feature of AE, with Tim-4+ macrophages identified as crucial mediators of this immunomodulatory effect. Our findings demonstrate that Tim-4 signaling facilitates metacestode progression during chronic *E. multilocularis* infection. The liver immune microenvironment in the middle and late stages of mice infected with *E. multilocularis* was established successfully *in vitro* and we found that Tim-4+macrophages suppressed the dominant expression of the proinflammatory response to promote parasite immune escape. Tim-4 expression promoted antigen-specific tumor tolerance in a mouse cancer model. Uptake of apoptotic cells via TIM4-expressing tumor-infiltrating macrophages led to activation of autophagic degradation and reduced tumor antigen presentation (Baghdadi et al., 2013).

As a result, Tim-4 emerged as a potential target associated with *E. multilocularis* infection in patients with hepatic AE. Parasitic infections impose a significant disease burden, characterized by hepatocyte destruction and subsequent release of damage-associated molecular patterns (DAMPs). These DAMPs trigger monocyte and macrophage recruitment to hepatic tissues. The resulting chronic inflammatory state drives progressive liver fibrosis, with both resident Kupffer cells (KCs) and recruited monocyte-derived macrophages demonstrated to contribute to this fibrotic progression (Wang et al., 2025). Perhaps the best recognized of the APCs in the liver are the KCs, which are the largest macrophage population in the body, representing 80%–90% of all tissue-resident macrophages (Chen et al., 2008). Although non-motile, KCs live directly within the bloodstream, residing within the lumen of the liver sinusoids, and providing optimal positioning for continuous immune surveillance, antigen sampling, and pathogen capture (Tian et al., 2024). Although KCs are poor stimulators of T-cell activation under basal conditions (Fratini et al., 2020), the presence of other pathogen-associated molecules or inflammatory cytokines can modulate the KCs, converting these normally tolerogenic cells into potent APCs capable of robust T-cell activation (Díaz et al., 2023). In a murine model of collagen-induced arthritis, it appears that anti-Tim-4 acted via inflammatory macrophages and osteoclasts, as no changes in T-cell function were observed later in the course of the disease (Abe et al., 2013).

Tim-4 deficiency or blockade was protective in liver ischemia-reperfusion injury (IRI) and was associated with decreased macrophage infiltration, decreased neutrophil infiltration, and decreased chemokine and proinflammatory cytokine (TNF-α, IL-1β, and IL-6) expression (Ji et al., 2014; Li et al., 2015). To explore the functional diversity of macrophages throughout *E. multilocularis* infection, we focused on the canonical macrophage-associated cytokines (IL6, IL‐18, IL‐1β, TNF-α, and IL‐10) that critically determine macrophage polarization and effector functions. Our findings demonstrate that these cytokines collectively orchestrate macrophage activation states during infection. Likewise, we observed that upregulated TNF-α expression correlated with monocyte apoptosis, which can prevent immune response to the *E. multilocularis* infection (Yang et al., 2012). An *in vivo* investigation has shown that increased levels of TLR2 and TLR4 mRNA expression and related cytokines (IFN-γ, IL-5, IL-23, and IL-10) in patients with hepatic AE protect the parasite from host immunity (Abudusalamu et al., 2016).

Wu et al. discovered that Tim-4 interference in the KCs reduced the TGF-β secretion during liver fibrosis (Wu et al., 2020). HSCs incubated with KCs from Tim-4 interference mice showed low expression levels of fibronectin and collagen 1α, which are required for HSC function. This is basically consistent with our results in the indirect co-culture cell model. The efficient phagocytic clearance of apoptotic cells is crucial for preventing secondary necrosis and the subsequent release of proinflammatory cellular contents. As a phosphatidylserine receptor, Tim-4 plays a pivotal role in this physiological process, which is fundamental for maintaining immune homeostasis and self-tolerance (McGrath, 2018). Apoptosis is a programmed cell death that is finely tuned according to the host-parasite interaction (Spotin et al., 2012). Apoptosis can reciprocally exert a bi-functional effect on the drug-*Echinococcus* parasite and the host-*Echinococcus* metabolite relationships in the suppressive and survival mechanisms of the parasite, respectively (Bakhtiar et al., 2020). Analyses of DNA fragmentation and caspase3 activity in the germinal layer confirm that apoptosis has a negative regulatory effect on the generation of PSCs and leads to possible infertility of hydatid cysts (Paredes et al., 2007). Our results also suggested that the apoptosis level of cells increased with high expression of Tim-4. Under appropriate conditions, hepatocytes can function as intrahepatic APCs (Zhou et al., 2022), detecting pathogens and presenting antigens to the adaptive immune system (Shojaie et al., 2024). Hepatocytes make up approximately 80% of all liver cells and express a wide variety of immune receptors (e.g., PRRs, MHCs, and costimulatory and adhesion molecules) (Huang et al., 2023). Although some of these receptors play a role in hepatocyte-mediated immunity (e.g., inhibition of viral replication, production and release of inflammatory cytokines, and acute-phase proteins), others activate and coordinate the adaptive immune response (Zhou et al., 2022). Although hepatocytes can directly support T cell activation and immunity against intracellular infections (e.g., viruses), given the surrounding environment rich in other, more “professional” APCs (e.g., KCs, DCs, LSECs) (Zhou et al., 2022), it remains unclear to what extent hepatocytes functionally contribute to generating immune responses to exogenous antigens.

There are some limitations in our study. Our study found that hepatocytes also expressed Tim-4. We hypothesized that Tim-4 is secreted by classical APCs, such as macrophages or DC cells, and then released to hepatocytes to play an immune regulation role. However, the partial hepatocyte itself plays an antigen-presenting role in the course of parasitic infection and autonomously secretes Tim-4 to play an immunosuppressive role, but further study is needed.

## Conclusions

In summary, this study indicated that Tim-4 suppressed the dominant expression of the proinflammatory response to promote parasite immune escape. Inhibition of Tim-4 expression on macrophages even reversed the level of liver fibrosis. Our research indicates the immune function of Tim-4 in parasite infection immunity, and it may provide some new ideas regarding *E. multilocularis* infection.

## Data Availability

All relevant data is contained within the article: The original contributions presented in the study are included in the article, further inquiries can be directed to the corresponding authors.

## References

[B1] Abe Y. Kamachi F. Kawamoto T. Makino F. Ito J. Kojima Y.. (2013). TIM-4 has dual function in the induction and effector phases of murine arthritis. J. Immunol. 191, 4562–4572. doi: 10.4049/jimmunol.1203035 24068667

[B2] Abudusalamu A. Tuerhongjiang T. Ma H. Z. Zhang H. Zhang H. Abudukaiyoumu M.. (2016). Changes of toll-like receptor mRNA and related cytokines in patients with hepatic alveolar echinococcosis. Zhongguo ji sheng chong xue yu ji sheng chong bing za zhi. 34, 542–546. 30141853

[B3] Akbulut S. Cicek E. Kolu M. Sahin T. T. Yilmaz S. (2018). Associating liver partition and portal vein ligation for staged hepatectomy for extensive alveolar echinococcosis: First case report in the literature. World. J. Gastrointest. Surg. 10, 1–5. doi: 10.4240/wjgs.v10.i1.1 29391928 PMC5785687

[B4] Baghdadi M. Yoneda A. Yamashina T. Nagao H. Komohara Y. Nagai S.. (2013). TIM-4 glycoprotein-mediated degradation of dying tumor cells by autophagy leads to reduced antigen presentation and increased immune tolerance. Immunity. 39, 1070–1081. doi: 10.1016/j.immuni.2013.09.014 24315994

[B5] Bakhtiar N. M. Spotin A. Mahami-Oskouei M. Ahmadpour E. Rostami A. (2020). Recent advances on innate immune pathways related to host-parasite cross-talk in cystic and alveolar echinococcosis. Parasites vectors. 13, 232. doi: 10.1186/s13071-020-04103-4 32375891 PMC7204293

[B6] Casulli A. (2020). Recognising the substantial burden of neglected pandemics cystic and alveolar echinococcosis. Lancet Glob Health 8, e470–e471. doi: 10.1016/S2214-109X(20)30066-8 32199112

[B7] Chen Y. Liu Z. Liang S. Luan X. Long F. Chen J.. (2008). Role of Kupffer cells in the induction of tolerance of orthotopic liver transplantation in rats. Liver Transpl. 14, 823–836. doi: 10.1002/lt.21450 18508376

[B8] Chow A. SChad S. Green M. D. Hellmann M. D. Allaj V. Ceglia N.. (2021). Tim-4(+) cavity-resident macrophages impair anti-tumor CD8(+) T cell immunity. Cancer Cell. 39, 973–988.e9. doi: 10.1016/j.ccell.2021.05.006 34115989 PMC9115604

[B9] Díaz Á. Barrios A. A. Grezzi L. Mouhape C. Jenkins S. J. Allen J. E.. (2023). Immunology of a unique biological structure: the Echinococcus laminated layer. Protein Cell. 14, 87–104. doi: 10.1093/procel/pwac023 36929004 PMC10019577

[B10] Dorfman D. M. Hornick J. L. Shahsafaei A. Freeman G. J. (2010). The phosphatidylserine receptors, T cell immunoglobulin mucin proteins 3 and 4, are markers of histiocytic sarcoma and other histiocytic and dendritic cell neoplasms. Hum. Pathol. 41, 1486–1494. doi: 10.1016/j.humpath.2010.04.005 20656318 PMC3115740

[B11] Fratini F. Tamarozzi F. Macchia G. Bertuccini L. Mariconti M. Birago C.. (2020). Proteomic analysis of plasma exosomes from Cystic Echinococcosis patients provides *in vivo* support for distinct immune response profiles in active vs inactive infection and suggests potential biomarkers. PLoS Negl. Trop. Dis. 14, e0008586. doi: 10.1371/journal.pntd.0008586 33017416 PMC7535053

[B12] Gao W. Jin Z. Zheng Y. Xu Y. (2021). Psoralen inhibits the inflammatory response and mucus production in allergic rhinitis by inhibiting the activator protein 1 pathway and the downstream expression of cystatin−SN. Mol. Med. Rep. 24, 652. doi: 10.3892/mmr.2021.12291 34278468 PMC8299190

[B13] Ghasemirad H. Bazargan N. Shahesmaeili A. Harandi M. F. (2022). Echinococcosis in immunocompromised patients: A systematic review. Acta Trop. 232, 106490. doi: 10.1016/j.actatropica.2022.106490 35490729

[B14] Gottstein B. Soboslay P. Ortona E. Wang J. Siracusano A. Vuitton D. A. (2017). Immunology of alveolar and cystic echinococcosis (AE and CE). Adv. Parasitol. 96, 1–54. doi: 10.1016/bs.apar.2016.09.005 28212788

[B15] Huang C. Zhou Y. Cheng J. Guo X. Shou D. Quan Y.. (2023). Pattern recognition receptors in the development of nonalcoholic fatty liver disease and progression to hepatocellular carcinoma: An emerging therapeutic strategy. Front. Endocrinol. (Lausanne). 14. doi: 10.3389/fendo.2023.1145392 PMC10067914 37020586

[B16] Jenkins S. J. Allen J. E. (2021). The expanding world of tissue-resident macrophages. Eur. J. Immunol. 51, 1882–1896. doi: 10.1002/eji.202048881 34107057

[B17] Ji H. Liu Y. Zhang Y. Shen X. D. Gao F. Busuttil R. W.. (2014). T-cell immunoglobulin and mucin domain 4 (TIM-4) signaling in innate immune-mediated liver ischemia-reperfusion injury. Hepatology. 60, 2052–2064. doi: 10.1002/hep.27334 25066922 PMC4396987

[B18] Joliat G. R. Martins-Filho S. N. Haefliger S. Demartines N. Halkic N. Labgaa I.. (2023). Programmed death-ligand1 is a determinant of recurrence in alveolar echinococcosis. Int. J. Infect. Dis. 129, 285–288. doi: 10.1016/j.ijid.2023.01.043 36775187

[B19] Kuninaka Y. Ishida Y. Ishigami A. Nosaka M. Matsuki J. Yasuda H.. (2022). Macrophage polarity and wound age determination. Sci. Rep. 12, 20327. doi: 10.1038/s41598-022-24577-9 36434083 PMC9700750

[B20] Lachenmayer A. Gebbers D. Gottstein B. Candinas D. Beldi G. (2019). Elevated incidence of alveolar echinococcosis in immunocompromised patients. Food Waterborne Parasitol. 16, e00060. doi: 10.1016/j.fawpar.2019.e00060 32095630 PMC7034048

[B21] Li J. Yang Y. Han X. Li J. Tian M. Qi W.. (2023). Oral delivery of anti-parasitic agent-loaded PLGA nanoparticles: enhanced liver targeting and improved therapeutic effect on hepatic alveolar echinococcosis. Int. J. Nanomedicine. 18, 3069–3085. doi: 10.2147/IJN.S397526 37312930 PMC10259527

[B22] Li J. Zhao X. Liu X. Liu H. (2015). Disruption of TIM-4 in dendritic cell ameliorates hepatic warm IR injury through the induction of regulatory T cells. Mol. Immunol. 66, 117–125. doi: 10.1016/j.molimm.2015.02.004 25771178

[B23] Ma T. Wang Q. Hao M. Xue C. Wang X. Han S.. (2023). Epidemiological characteristics and risk factors for cystic and alveolar echinococcosis in China: an analysis of a national population-based field survey. Parasit Vectors. 16, 181. doi: 10.1186/s13071-023-05788-z 37270512 PMC10239570

[B24] McGrath M. M. (2018). Diverse roles of TIM4 in immune activation: implications for alloimmunity. Curr. Opin. Organ Transplant. 23, 44–50. doi: 10.1097/MOT.0000000000000487 29189411

[B25] Meng R. Fu Y. Zhang Y. Mou Y. Liu G. Fan H. (2022). Indoleamine 2,3-dioxygenase 1 signaling orchestrates immune tolerance in Echinococcus multilocularis-infected mice. Front. Immunol. 13. doi: 10.3389/fimmu.2022.1032280 PMC9691980 36439161

[B26] Miyanishi M. Tada K. Koike M. Uchiyama Y. Kitamura T. Nagata S. (2007). Identification of Tim4 as a phosphatidylserine receptor. Nature. 450, 435–439. doi: 10.1038/nature06307 17960135

[B27] Pang N. Zhang F. Ma X. Zhang Z. Zhao H. Xin Y.. (2014). Th9/IL-9 profile in human echinococcosis: their involvement in immune response during infection by Echinococcus granulosus. Mediators Inflamm. 2014, 781649. doi: 10.1155/2014/781649 24799769 PMC3985320

[B28] Paredes R. Jiménez V. Cabrera G. Iragüen D. Galanti N. (2007). Apoptosis as a possible mechanism of infertility in Echinococcus granulosus hydatid cysts. J. Cell Biochem. 100, 1200–1209. doi: 10.1002/jcb.21108 17031852

[B29] Shojaie L. Bogdanov J. M. Alavifard H. Mohamed M. G. Baktash A. Ali M.. (2024). Innate and adaptive immune cell interaction drives inflammasome activation and hepatocyte apoptosis in murine liver injury from immune checkpoint inhibitors. Cell Death Dis. 15, 140. doi: 10.1038/s41419-024-06535-7 38355725 PMC10866933

[B30] Spotin A. Majdi M. M. Sankian M. Varasteh A. (2012). The study of apoptotic bifunctional effects in relationship between host and parasite in cystic echinococcosis: a new approach to suppression and survival of hydatid cyst. Parasitol Res. 110, 1979–1984. doi: 10.1007/s00436-011-2726-4 22167369

[B31] Takagi-Kimura M. Tada A. Kijima T. Kubo S. Ohmuraya M. Yoshikawa Y. (2022). BAP1 depletion in human B-lymphoblast cells affects the production of innate immune cytokines and chemokines. Genes Cells 27, 731–740. doi: 10.1111/gtc.12988 36300836

[B32] Tian F. Jiang T. Qi X. Zhao Z. Li B. Aibibula M.. (2021). Role of cytokines on the progression of liver fibrosis in mice infected with echinococcus multilocularis. Infect. Drug Resist. 14, 5651–5660. doi: 10.2147/IDR.S344508 34992391 PMC8714463

[B33] Tian F. Liu Y. Gao J. Yang N. Shang X. Lv J.. (2020). Study on the association between TGF-beta1 and liver fibrosis in patients with hepatic cystic echinococcosis. Exp. Ther. Med. 19, 1275–1280. doi: 10.3892/etm.2019.8355 32010299 PMC6966196

[B34] Tian X. Wang J. Chen H. Ding M. Jin Q. Zhang J. R. (2024). *In vivo* functional immunoprotection correlates for vaccines against invasive bacteria. Vaccine. 42, 853–863. doi: 10.1016/j.vaccine.2024.01.018 38233287

[B35] Wang L. Y. Qin M. Liu Z. H. Wu W. P. Xiao N. Zhou X. N.. (2021). Prevalence and spatial distribution characteristics of human echinococcosis in China. PLoS Negl. Trop. Dis. 15, e0009996. doi: 10.1371/journal.pntd.0009996 34962928 PMC8789093

[B36] Wang Y. Wang P. Yu Y. Huang E. Yao Y. Guo D.. (2023). Hepatocyte Ninjurin2 promotes hepatic stellate cell activation and liver fibrosis through the IGF1R/EGR1/PDGF-BB signaling pathway. Metabolism. 140, 155380. doi: 10.1016/j.metabol.2022.155380 36549436

[B37] Wang H. Zhang C. S. Fang B. B. Hou J. Li W. D. Li Z. D.. (2020). Dual role of hepatic macrophages in the establishment of the echinococcus multilocularis metacestode in mice. Front. Immunol. 11. doi: 10.3389/fimmu.2020.600635 PMC7820908 33488594

[B38] Wang L. Zhu J. Meng M. Zhu S. Ma Y. Zhou T.. (2025). Inhibition of the MyD88/NF-kappaB pathway alters the Th1/Th2 balance to exacerbate liver injury and hepatic fibrosis in alveolar echinococcosis. FASEB J. 39, e70472. doi: 10.1096/fj.202402423RR 40116193

[B39] Wen H. Vuitton L. Tuxun T. Li J. Vuitton D. A. Zhang W.. (2019). Echinococcosis: advances in the 21st century. Clin. Microbiol Rev. 32, e00075–e00018. doi: 10.1128/CMR.00075-18 30760475 PMC6431127

[B40] Wu H. Chen G. Wang J. Deng M. Yuan F. Gong J. (2020). TIM-4 interference in Kupffer cells against CCL4-induced liver fibrosis by mediating Akt1/Mitophagy signalling pathway. Cell Prolif. 53, e12731. doi: 10.1111/cpr.12731 31755616 PMC6985653

[B41] Yang H. Q. Ma S. B. Bian Z. Y. Li J. Zou H. Zhang S. J.. (2012). Expression of tumor necrosis factor-alpha and caspase-3 protein in monocytes adjacent to the invaded Echinococcus multilocularis in liver. Zhongguo Ji Sheng Chong Xue Yu Ji Sheng Chong Bing Za Zhi 30, 201–205. 23072136

[B42] Zhang C. Wang L. Ali T. Li L. Bi X. Wang J.. (2016). Hydatid cyst fluid promotes peri-cystic fibrosis in cystic echinococcosis by suppressing miR-19 expression. Parasit Vectors. 9, 278. doi: 10.1186/s13071-016-1562-x 27177776 PMC4866024

[B43] Zhou Y. Zhang H. Yao Y. Zhang X. Guan Y. Zheng F. (2022). CD4(+) T cell activation and inflammation in NASH-related fibrosis. Front. Immunol. 13. doi: 10.3389/fimmu.2022.967410 PMC9399803 36032141

